# “Urate and NOX5 Control Blood Digestion in the Hematophagous Insect *Rhodnius prolixus*”

**DOI:** 10.3389/fphys.2021.633093

**Published:** 2021-02-25

**Authors:** Ana Caroline P. Gandara, Felipe A. Dias, Paula C. de Lemos, Renata Stiebler, Ana Cristina S. Bombaça, Rubem Menna-Barreto, Pedro L. Oliveira

**Affiliations:** ^1^Instituto de Bioquímica Médica Leopoldo de Meis, Universidade Federal do Rio de Janeiro, Rio de Janeiro, Brazil; ^2^Instituto Oswaldo Cruz, Rio de Janeiro, Brazil; ^3^Instituto Nacional de Ciência e Tecnologia em Entomologia Molecular, Rio de Janeiro, Brazil

**Keywords:** urate, NADPH oxidase, ROS, blood digestion, *Rhodnius prolixus*

## Abstract

Low levels of reactive oxygen species (ROS) are now recognized as essential players in cell signaling. Here, we studied the role of two conserved enzymes involved in redox regulation that play a critical role in the control of ROS in the digestive physiology of a blood-sucking insect, the kissing bug *Rhodnius prolixus*. RNAi-mediated silencing of *RpNOX5* and *RpXDH* induced early mortality in adult females after a blood meal. Recently, a role for *RpNOX5* in gut motility was reported, and here, we show that midgut peristalsis is also under the control of *RpXDH*. Together with impaired peristalsis, silencing either genes impaired egg production and hemoglobin digestion, and decreased hemolymph urate titers. Ultrastructurally, the silencing of *RpNOX5* or *RpXDH* affected midgut cells, changing the cells of blood-fed insects to a phenotype resembling the cells of unfed insects, suggesting that these genes work together in the control of blood digestion. Injection of either allopurinol (an XDH inhibitor) or uricase recapitulated the gene silencing effects, suggesting that urate itself is involved in the control of blood digestion. The silencing of each of these genes influenced the expression of the other gene in a complex way both in the unfed state and after a blood meal, revealing signaling crosstalk between them that influences redox metabolism and nitrogen excretion and plays a central role in the control of digestive physiology.

## Introduction

Reactive oxygen species (ROS) control many processes, from gene expression and protein translation to metabolism and cell signaling, and the NAD, NADP and thiol/disulfide systems are important for ROS signaling (Jones and Sies, 2015). ROS are produced in different parts of the cell and modulate key target functions *via* the oxidative modification of redox-sensitive essential proteins and alterations in redox homeostasis that are associated with many different disease conditions (Holmström and Finkel, 2014; Sies, 2017; Bardaweel et al., 2018).

NADPH oxidases (NOXes) are one of the major sources of cellular ROS, and they are still the focus of extensive research interest due to their exclusive function in producing ROS under normal physiological conditions. Arthropods have three NOX types (Gandara et al., 2017): NOX4-art, an arthropod-specific p22-*phox*-independent NOX4, and two calcium-dependent enzymes, DUOX, which produces hydrogen peroxide (De Deken et al., 2002; Dias et al., 2013), and NOX5, which produces superoxide (Bánfi et al., 2001; Montezano et al., 2018). In the gut, DUOX-dependent ROS production from bacteria-stimulated *Drosophila melanogaster* mucosa is an important pathogen-killing mechanism (Ha et al., 2005) and can increase defecation as a defense response (Du et al., 2016). *Rhodnius prolixus* only has calcium-activated DUOX and NOX5, which are involved in eggshell hardening and gut motility, respectively, in this insect (Dias et al., 2013; Montezano et al., 2018).

Xanthine dehydrogenase (XDH) is widely distributed in metazoan organisms and catalyzes the reduction of NAD coupled to the successive oxidation of hypoxanthine to xanthine and xanthine to urate (Wang et al., 2016), in accordance with urate being the main nitrogen metabolism end-product of insects (Wigglesworth, 1931; Barrett and Friend, 1966, 1970; Briegel, 1986). In addition to this key role in nitrogen excretion, urate is an important regulator of redox balance (Hilliker et al., 1992). In *R. prolixus*, urate is the major antioxidant present in the hemolymph, where high concentrations (>5 mM) are attained after a blood meal (Souza et al., 1997).

The most canonical signaling pathway in the regulation of digestion in blood-feeding insects acts through the activation of neurons and endocrine cells in response to mechanical stimuli or nutrients (Thomsen and Muller, 1959; Maddrell, 1964; Persaud and Davey, 1971; Maddrell and Gardiner, 1975; Cole and Gillett, 1979; Houseman and Downe, 1983; Wu et al., 2020). The ingested blood induces proteinase production and nutrient uptake (Garcia and Garcia, 1977; Sanders et al., 2003; Henriques et al., 2017) to promote its own retention in the midgut (Caroci and Noriega, 2003) and to control ROS production in the gut (Oliveira et al., 2011; Gandara et al., 2016). The anterior midgut (AM) is involved in hemolysis, ion and water transport, carbohydrate digestion and glycogen and lipid storage (Billingsley, 1990; Vieira et al., 2015; Henriques et al., 2017). Once hemolysis starts in the AM (De Azambuja et al., 1983), hemoglobin and other proteins start being sent to the posterior midgut (PM), which stimulates proteinase activity (Garcia and Garcia, 1977). In the PM, high proteinase activity produces amino acids and heme (as byproducts of hemoglobin degradation), which are released to the hemolymph (Wigglesworth, 1943; Barrett, 1974) to be delivered to the ovaries for the production of yolk proteins during vitellogenesis (Atella et al., 2005). However, the cellular and molecular mechanisms underlying these sensory and physiological functions are poorly understood (LaJeunesse et al., 2010; Du et al., 2016).

In recent years, a significant research effort has focused on the function of intestinal ROS (Aviello and Knaus, 2018), in most cases, in gut immunity. Relatively little is known about the role of ROS in digestion and how host metabolic factors control the redox state and redox signaling. Blood-sucking insects ingest blood meals that are several-fold their weights before feeding. Vertebrate blood is comprised of 85% protein (dry weight), and the most abundant protein is hemoglobin. Therefore, these animals present a unique situation regarding nitrogen and redox metabolism, as large amounts of both amino acids and heme (a pro-oxidant molecule) are produced during the digestion of a blood meal (Graça-Souza et al., 2006; Sterkel et al., 2017). Here, while studying the roles of a ROS-producing enzyme (*RpNOX5*) and an antioxidant-producing enzyme (*RpXDH*), we identified blood ingestion-induced signaling mechanisms associated with changes in redox metabolism in a physiology insect model, the triatomine *R. prolixus*.

## Materials and Methods

### Insects and Ethics Statement

All animal care and experimental protocols were conducted in accordance with the guidelines of the Committee for Evaluation of Animal Use for Research (Universidade Federal do Rio de Janeiro, CAUAP-UFRJ) and the NIH Guide for the Care and Use of Laboratory Animals (ISBN 0-309-05377-3). Protocols were approved by CAUAP-UFRJ under registry #IBQM 149-9. Dedicated technicians in the animal facility at the Instituto de Bioquímica Médica Leopoldo de Meis (UFRJ) carried out all protocols related to rabbit husbandry under strict guidelines, with supervision of veterinarians to ensure appropriate animal handling. *R. prolixus* colony was maintained at 28°C and 70–80% relative humidity, and the insects used were adult mated females fed rabbit blood at 3-week intervals.

### Identification of the *RpNOX5* and *RpXDH* Genes

A local BLAST search using the cDNA sequences of NOX5 and XDH as queries was used to identify NOX5 and XDH sequences in the *R. prolixus* transcriptome (Ribeiro et al., 2014). These partial cDNAs were used to identify the full-length transcripts of NOX5 (RPRC008329) and XDH (RPRC011533) in the *R. prolixus* genome database available at VectorBase (Version RproC3) (Mesquita et al., 2015).

### *RpNOX5* and *RpXDH* Structures

Transmembrane α-helices were predicted using the TMHMM server v.2.0, available from the Center for Biological Sequence Analysis, and the amino acid residue hydrophobicity profile was analyzed (Kyte and Doolittle, 1982). The alignments were performed with ClustalW software (Larkin et al., 2007), using the following sequences: *Acyrthosiphon pisum* (NOX5, Gene ID 328705704 and XDH, Gene ID 328699235), *Anopheles gambiae* (NOX5, Gene ID 158297105 and XDH, Gene ID 118789655), *Drosophila melanogaster* (NOX5, Gene ID 161077140 and XDH, Gene ID 17737937) and *Homo sapiens* (NOX5, Gene ID 74717091 and XDH, Gene ID 91823271). The positions of conserved domains and residues were identified at Pfam^1^ and edited in GeneDoc (Nicholas and Nicholas, 1997).

### RNA Extraction, Conventional PCR, and qPCR

Total RNA was extracted from tissues using TRIzol (Invitrogen) according to the manufacturer’s protocol. RNA was treated with RNase-free DNase I (Fermentas International Inc., Canada), and cDNA was synthesized using the High-Capacity cDNA Reverse Transcription kit (Applied Biosystems, United States). cDNA from the salivary glands, heart, Malpighian tubules, anterior midgut, posterior midgut, hindgut, fat body, cerebrum, flight muscle and ovary was PCR-amplified using PCR master mix (Fermentas International Inc., Canada), and the same primers were used for qPCR (described below). The fragments were separated by agarose gel electrophoresis (2% w/v), and their sizes were compared with GeneRulerTM 100 bp Plus DNA ladder fragments (Fermentas International Inc., Canada). qPCR was performed on a StepOnePlus real-time PCR system (Applied Biosystems, United States) using Power SYBR Green PCR master mix (Applied Biosystems, United States). The comparative Ct method (Livak and Schmittgen, 2001) was used to compare gene expression levels. The *R. prolixus EF-1 S* rRNA gene (RPRC015041) was used as an endogenous control (Majerowicz et al., 2011). The primer pairs used for the amplification of the NOX5, XDH and EF-1 cDNA fragments for both conventional and real-time PCR, named NOX5Rt, XDHRt and EF1Rt, respectively, are described in Supplementary Table 1.

### RNAi Experiments

To investigate the function of *RpXDH* and *RpNOX5*, gene silencing was performed by injecting dsRNA into the insect hemocoel. A 457-bp fragment from the *RpNOX5* gene and a 461-bp fragment from the *RpXDH* gene were amplified from reverse-transcribed RNAs extracted from *R. prolixus* tissues using the primer pairs NOX5Ds1 and XDHDs1, respectively. The amplification products were subjected to nested PCR with an additional pair of primers (NOX5Ds2 and XDHDs2) that included the T7 promoter sequence in each fragment. The primers mentioned above are described in Supplementary Table 1. The nested PCRs generated 497-bp and 444-bp fragments of *RpNOX5* and *RpXDH*, respectively. These fragments were used as a template to synthesize double-stranded RNA (dsRNA) specific for *RpNOX5* (dsNOX5) and *RpXDH* (dsXDH) using the MEGAscript RNAi kit (Ambion, United States) according to the manufacturer’s protocol. An unrelated dsRNA (dsMal) specific for the *Escherichia coli* MalE gene (Gene ID 948538) was used as a control for the off-target effects of dsRNA. The Mal fragment was amplified from the Litmus 28i-mal plasmid (New England Biolabs, United States) with a single primer (T7, 5-TAATACGACTCACTATAGGG-3) specific for the T7 promoter sequence that is on both sides of the MalE sequence. 21–22 days after the first blood feeding as adults, females were injected in the hemocoel with 1 μL of sterile distilled water containing 1 mg/mL dsRNA using a 5 μL Hamilton syringe. Six days after dsRNA injection, the insects were fed rabbit blood (blood-fed condition) or just dissected (unfed condition).

### Images and Video Acquisition of Gut Contractions

Insects were dissected 7 days after a blood meal (ABM), and the midguts were photographed. After removing the wings and legs, water drops were placed on the dorsal surface of the abdominal cuticle to improve the visualization of the movements of internal organs in the live insects, and 2-minute videos were acquired, as previously published (Montezano et al., 2018). Squares containing areas with partial visualization of the AM close to an ovary were selected. Using the Multimeasure option in ImageJ, the ratio of the dark areas (AM) to the white areas (ovary) was extracted and plotted against time to evaluate peristaltic movement (referred to as peristaltic contraction amplitude).

### Hemoglobin Quantification

AMs were dissected just after the blood meal (between 0.5–2 h), 4 days and 7 days ABM and homogenized in 250 μL of PBS (10 mM Na–phosphate, 0.15 M NaCl, pH 7.4). Hemoglobin content was assayed with a colorimetric kit (K023 kit, Bioclin, Brazil) based on the Drabkin method (Drabkin and Austin, 1935), according to the manufacturer’s protocol. The absorbance of the supernatants was read at 540 nm with a plate spectrophotometer (Spectramax M3, Molecular Devices, United States).

### Heme Quantitation

Quantitation of total heme was performed by the alkaline pyridine method (Falk, 1964) using the extinction values of the subtraction spectra (reduced-oxidized heme spectra). The posterior midguts and hindguts were collected individually and homogenized in 1 mL of 0.1 M NaOH. The tubes were centrifuged for 10 min at 15,000 × *g*, and the supernatants were collected. An aliquot of 10 μL of supernatant was added to 190 μL of 0.1 M NaOH, 200 μL of distilled water and 500 μL of alkaline pyridine solution [20% (v/v) 1 M NaOH, 48% (v/v) pyridine, 32% (v/v) water]. Visible light spectra were collected (500–600 nm) using a Shimadzu UV-2550 spectrophotometer (Japan) before and after the samples were reduced with sodium dithionite.

### Urate Quantification

Hemolymph (3 μL) was collected 4 days ABM, in the presence of phenylthiourea, diluted with 12 μL of ultrapure water and used immediately for assays in 96-well plates, according to the guidelines of the colorimetric kit (Doles, Brazil), based on the uricase reaction coupled to H_2_O_2_ 4-aminoantipyrine oxidation by peroxidase (Barham and Trinder, 1972). Samples were incubated at 37°C for 30 min and read at 520 nm with a plate spectrophotometer (Spectra Max M3, Molecular Devices, United States).

### *Ex vivo* ROS Microscopy Assays

As previously described (Gandara et al., 2016), the wings, legs and dorsal plaques were removed. Initially, to assess ROS levels, the hemolymph was replaced with a 50 μM solution of the oxidant-sensitive fluorophore dihydroethidium (hydroethidine, DHE) (Invitrogen, United States) in L15 medium culture (Gibco, United States) containing 5% (v/v) fetal bovine serum. The samples were incubated in the dark at 28°C for 20 min. Then, the midguts were washed with 0.15 M NaCl and immediately transferred to a glass slide for fluorescence microscopy analysis. Quantitative evaluation of fluorescence was performed using a 20 X objective and 100-ms exposure with a Zeiss Observer Z1 (Germany) with a Zeiss Axio Cam MrM Zeiss. The data were analyzed using AxioVision version 4.8 software. The #15 filter set (excitation BP, 546/12 nm; beam splitter FT, 580 nm; emission LP, 590 nm) was used for DHE labeling.

### Transmission Electron Microscopy

*Rhodnius prolixus* AMs and PMs were dissected without feeding or 4 days ABM and fixed with 2.5% glutaraldehyde in 0.1 M Na-cacodylate buffer (pH 7.2) at room temperature for 1 h at 25°C and postfixed with a solution of 1% OsO_4_, 0.8% potassium ferricyanide and 2.5 mM CaCl_2_ in the same buffer for 1 h at 25°C. The samples were dehydrated in an ascending acetone series and embedded in five steps in PolyBed 812 resin (1:3, 1:1, 1:2, 2:3, and pure resin). Ultrathin sections were stained with uranyl acetate and lead citrate and examined with a Jeol JEM1011 transmission electron microscope (Japan) at Plataforma de Microscopia Eletrônica in Fundação Oswaldo Cruz.

### Allopurinol and Uricase Injections

Allopurinol (Sigma, United States) or uricase (Sigma, United States) was dissolved in *R. prolixus* physiological saline containing 130 mM NaCl, 8.6 mM KCl, 8.3 mM MgCl_2_, 10.2 mM NaHCO_3_, 4.3 mM Na_2_HPO_4_, 34 mM glucose, and 2 mM CaCl_2_ (Maddrell, 1969). The allopurinol solution needed to be heated at 50°C in a dry bath for several minutes to ensure complete dissolution. Three days before the blood meal, unfed insects were injected with 40 μg of allopurinol or 1 U of uricase using a Hamilton syringe, and the insects were dissected 4 days ABM. Control insects received *R. prolixus* saline only.

### Intestinal Microbiota Evaluation

Whole homogenates of individual AM or PM were serially diluted, plated on BHI agar and kept at 28°C for 7 days, after which the number of colonies was evaluated. In insects from our colony, *Rhodococcus rhodnii* is the only cultivable bacterium that grows on culture plates.

### Statistical Analysis

All experiments were repeated at least twice, and statistical analyses were performed using GraphPad Prism.

## Results

### Structural Features and Domains of *RpXDH* and *RpNOX5*

*R. prolixus* NOX5 and XDH, hereafter called *RpNOX5* and *RpXDH*, respectively, were initially found in a digestive apparatus transcriptome analysis (Ribeiro et al., 2014), and full-length predicted CDSs were identified in the genome (Mesquita et al., 2015) (Supplementary Figures 1, 2). Supplementary Figure 3 shows the characteristic domains (EF-hand, transmembrane and NOX domains) of *RpNOX5*, which are conserved among all NOX5 orthologs (Kawahara et al., 2007) and *RpXDH* also has all canonical XDH domains.

### *RpNOX5* or *RpXDH* Silencing Induces Early Mortality

**FIGURE 1:**
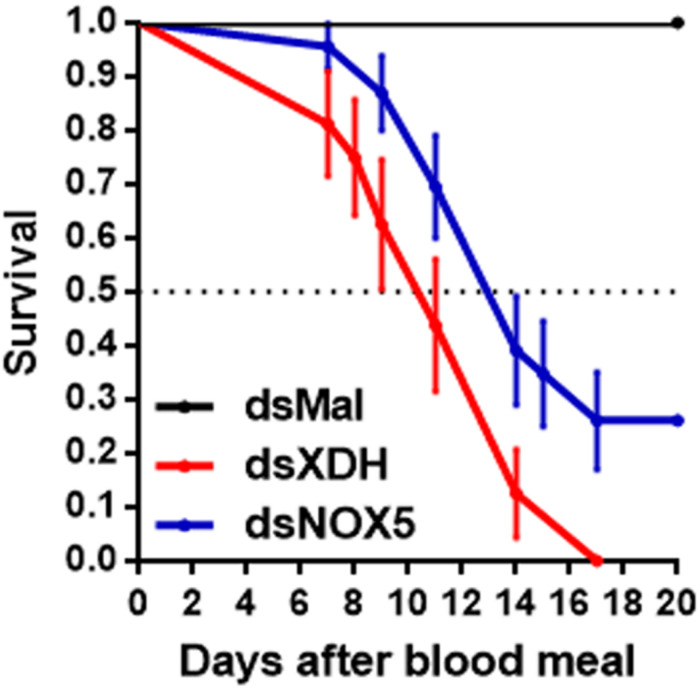
*RpNOX5* or *RpXDH* silencing induces early mortality in adult *R. prolixus* after a blood meal. Insects were fed rabbit blood, and dead individuals were counted daily. The horizontal dotted line represents 50% survival (*n* = 16–23 insects). *P* < 0.0001 for dsXDH- and dsNOX5-injected animals compared with dsMal-injected animals. Mantel-Cox and Gehan-Breslow-Wilcoxon test. The data represent the mean ± SEM.

*RpXDH* and *RpNOX5* mRNA were expressed in all tissues tested (Supplementary Figure 4). Efficient *RpNOX5* silencing was achieved before blood meal (by six days after *RpNOX5* dsRNA injection) but this effect was transient, and *RpNOX5* expression level recovered to control values at 4 days ABM). *RpXDH* silencing was achieved only by 4 days after a blood meal (Supplementary Figure 5). However, both *RpNOX5* or *RpXDH* silencing by RNAi induced early mortality in blood-fed adult females (Figure 1), revealing essential roles of both enzymes.

**FIGURE 2:**
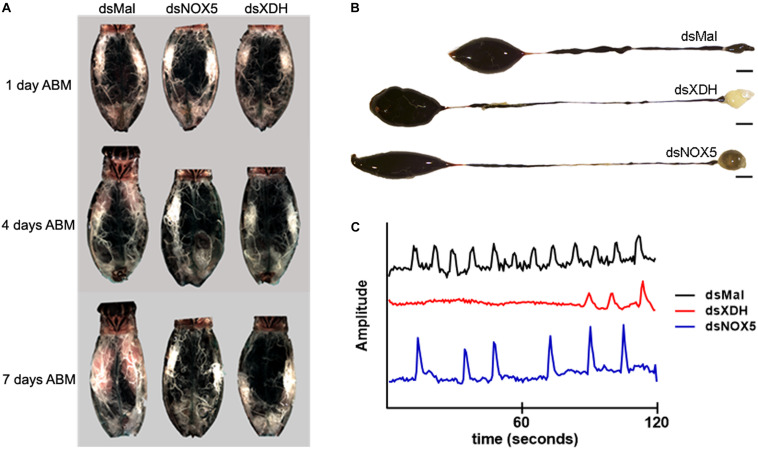
*RpNOX5* and *RpXDH* gene products affect midgut peristalsis. Insects were dissected 1, 4-, or 7-days ABM, and the organs were imaged. **(A)** The ovaries of silenced insects did not develop eggs. **(B)** Blood digestion inhibition is evidenced by reduced PM content in silenced insects compared to the control (dsMal). Scale bar: 30 mm. **(C)** The AM peristalsis pattern is altered, and the frequency of contraction is reduced in silenced insects. Representative traces of the relative amplitude of midgut contraction in a time interval of 2 min are shown. The mean frequency of contraction peaks (peaks/2 min, mean ± SEM) were 11.57 ± 1.21 (dsMal), 4.0 ± 1.45 (dsXDH) and 5.32 ± 1.21 (dsNOX5) (14–27 insects).

### *RpNOX5* or *RpXDH* Silencing Impairs Blood Digestion

**FIGURE 3:**
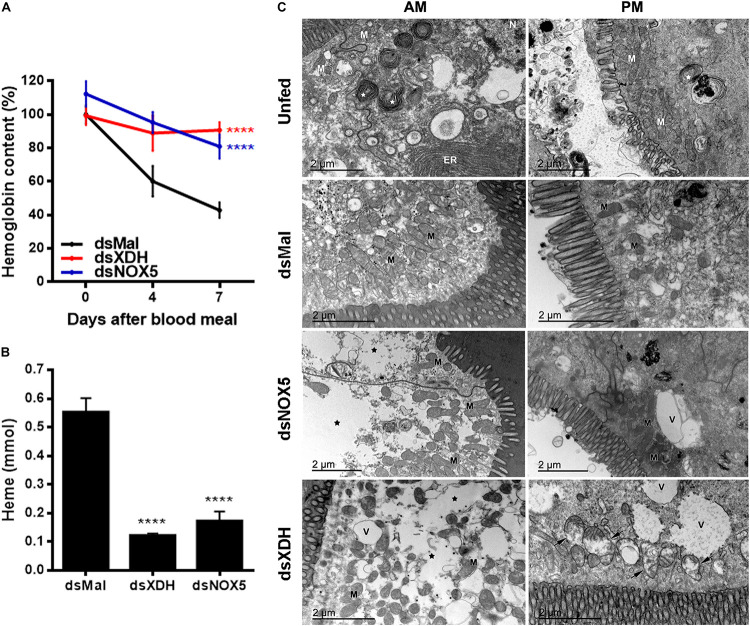
Silencing of *RpNOX 5* or *RpXDH* impairs blood digestion. **(A)** Data show the hemoglobin content of the AM (100% is the hemoglobin content of the AM of control insects (dsMal) 2 h ABM). Insects with gene silencing have more hemoglobin than controls at 7 days ABM (11–24 insects). **(B)** Data show the heme content of the PM (a product of blood digestion) 4 days ABM. dsXDH- and dsNOX5-injected animals presented less heme in the PM than did control insects (9–11 insects per condition). **(C)** AM and PM were dissected before receiving a blood meal (unfed) or 4 days ABM and processed for TEM. *RpNOX5* or *RpXDH* silencing causes cell damage in the gut, with decreased cell density in the AM and mitochondrial injury in the PM. ER, endoplasmic reticulum; V, vacuoles; M, mitochondria; white star, autophagy; black star, loss of cytosolic density; black arrow,: mitochondrial collapse with washed out aspect and loss of cristae as well as the formation of concentric membranar structures inside the organelle (9–24 insects). ****P < 0.0001, compared with dsMal-injected animals. One-way ANOVA and Dunnett’s or Dunn’s multiple comparisons posttest were used. The data represent the mean ± SEM.

*RpXDH or RpNOX5* silencing impaired egg development (almost no eggs were laid by silenced insects – data not shown) (Figure 2A) and AM peristalsis (Figure 2C). We also observed reduced PM content and very commonly the hindgut was enlarged and with a yellowish content in silenced animals (Figure 2B), but diuresis was not evaluated. In triatomine insects, no blood digestion occurs in the AM; blood proteins are kept undigested in the AM and are gradually transferred to the PM, where protein degradation is accomplished by means of cysteinic and aspartic proteinases, resulting in the release of heme from hemoglobin (Houseman and Downe, 1982). The silencing of *RpXDH or RpNOX5* produced a marked decrease in blood meal digestion, as revealed both by using the progressive reduction in hemoglobin content of the AM as a proxy for the pace of blood meal digestion (Figure 3A) or accumulation of heme in the PM and hindgut (Figure 3B). Overall, virtually identical phenotypes were obtained by the individual silencing of *RpXDH* or *RpNOX5*, suggesting that these genes are involved in the control of blood digestion.

### *RpNOX5* or *RpXDH* Silencing Causes Cell Damage in Gut

Transmission electron microscopy (TEM) analysis showed that unfed guts are stalled in an apparent autophagy-related structures that are reversed by a blood meal, as shown in the control animals (dsMal) in Figure 3C. However, silencing of *RpXDH* or *RpNOX5* led to a similar autophagic phenotype, characterized by loss of cytosolic density, increases in the size and number of autophagosomes, and a recurrent mitochondrial damage, with washed out matrix aspect, loss of cristae, and the presence of concentric membranar structures inside the organelle.

### *RpNOX5* or *RpXDH* Silencing Results in Altered ROS Levels Without Affecting the Gut Microbiota

**FIGURE 4:**
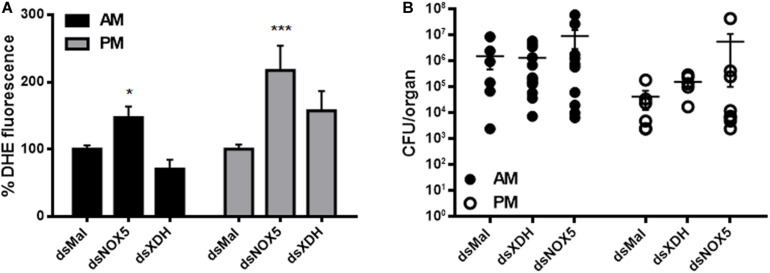
ROS levels were increased in *RpNOX5*-silenced animals without changes in the gut microbiota. **(A)** Midguts of insects dissected 4 days ABM were incubated with 50 μM DHE, and the fluorescence images were analyzed (8–20 insects). *P < 0.05, ***P < 0.0001 compared with dsMal-injected animals. ANOVA and Dunnett’s test were used to compare dsMal-injected animals. The data represent the mean ± SEM. **(B)** Evaluation of the cultivable population of *Rhodococcus rhodnii* from blood-fed midguts dissected 4 days ABM (5–13 insects). No significant differences were found. Kruskal-Wallis test, Dunn’s multiple comparisons posttest, compared with dsMal-injected animals. AM, anterior midgut; PM, posterior midgut.

As both enzymes are involved in the regulation of redox metabolism, ROS levels were evaluated by means of DHE fluorescence, an oxidant-sensitive probe. Unexpectedly, oxidant levels were higher in both the AM and PM of insects silenced for *RpNOX5*, a ROS-producing enzyme (Figure 4A). In contrast, insects silenced for *RpXDH* (an antioxidant-producing enzyme) showed only a trend (without statistical difference) of upregulation in ROS levels in the PM, compared with control insects (Figure 4A). As ROS production has been implicated in the control of both pathogens and indigenous microbiota (Ha et al., 2005; Oliveira et al., 2011), we evaluated the effect of *RpNOX5* or *RpXDH* silencing on the *R. prolixus* microbiota, which is known to be dominated by a mutualist symbiont, *R. rhodnii* (Baines, 1956). Despite the increase in ROS levels with *RpNOX5* silencing (Figure 4A), the results showed no statistically significant change in the bacterial population in *RpNOX5*-silenced insects compared to the control insects, although we did observe a trend toward an increase in the bacterial population, especially in the AM (Figure 4B).

### Urate-Mediated Regulation of Blood Digestion

**FIGURE 5:**
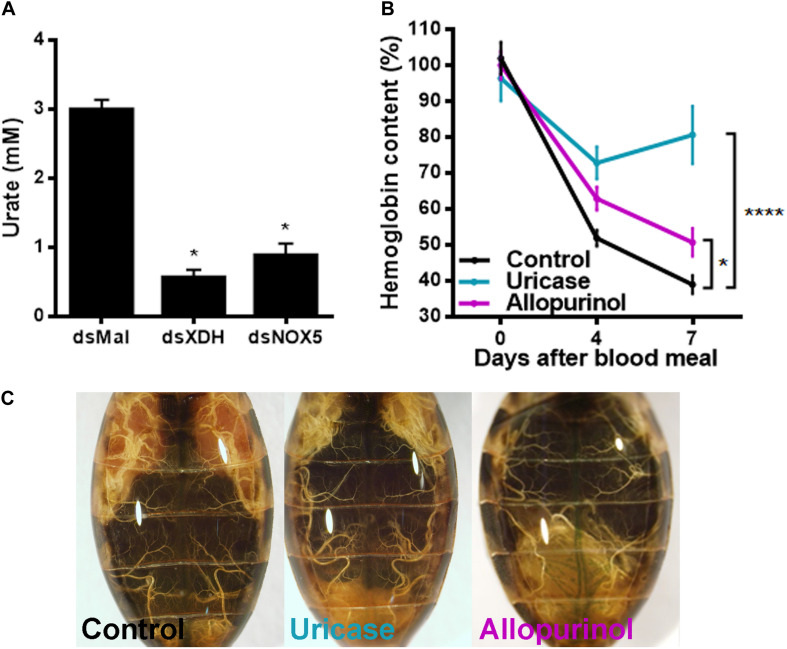
Allopurinol and uricase injection reproduces the phenotype induced by *RpXDH* RNAi. **(A)** Urate concentration in the hemolymph (16 insects per condition). **(B)** Hemoglobin content in anterior midguts. Three days before a blood meal, unfed insects were injected with 40 μg of allopurinol or 1 U of uricase into the hemocoel. Control insects received 10 μL of *R. prolixus* saline (8–49 insects). **(C)** Representatives images of injected animals. *P < 0.05; ****P < 0.0001 compared with dsMal-injected animals **(A)** or saline-injected animals **(B)**. One-way ANOVA and Dunnett’s multiple comparisons posttest were used. The data represent the mean ± SEM.

To test whether urate itself could be one of the signals involved in the control of blood digestion, insects were treated with allopurinol, an XDH inhibitor (Elion et al., 1963), or were injected with uricase to enzymatically reduce circulating urate levels. Urate titers in hemolymph decreased in insects with *RpXDH* silencing, as expected but were also markedly reduced after *RpNOX5* silencing (Figure 5A). Injection of allopurinol or uricase in the hemocoel 3 days before a blood meal closely recapitulated the gene silencing phenotype (Figure 2A), inhibiting blood digestion (Figure 5B) and egg production (Figure 5C).

### *RpNOX5* and *RpXDH* Reciprocal Transcriptional Control

**FIGURE 6:**
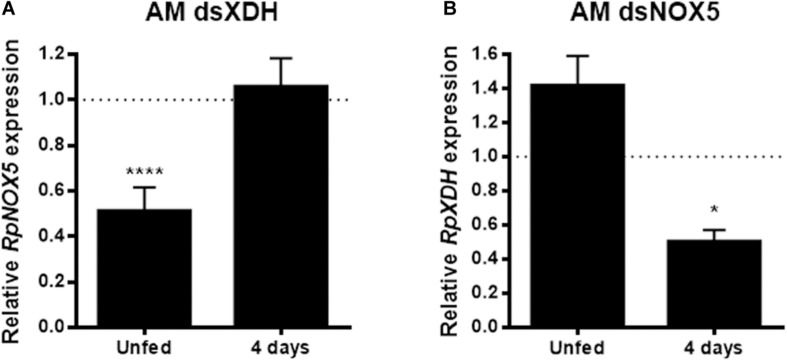
*RpNOX5* and *RpXDH* gene products interfere with each other’s expression levels. **(A)** *RpNOX5* and **(B)** *RpXDH* expression levels in the anterior midgut (AM). qPCR assays were performed with individual AMs dissected 6 days after dsRNA injection (unfed) or 4 days ABM. Expression levels were normalized to the level detected in the dsMal-injected animals (9–23 insects). *P < 0.0001 compared with dsMal-injected animals. Unpaired *t*-test. The data represent the mean ± SEM.

In the AM, *RpNOX5* showed reduced levels in the unfed insects and approached control levels at 4 days ABM (Supplementary Figure 5A). This transient silencing observed suggests that the signaling action of *RpNOX5* occurs before the 4th day ABM. The interruption of the signaling pathway by RNAi before the canonical signaling provoked by the arrival of blood in the midgut, was sufficient to prevent the triggering of the peristalsis/digestion process and generate a resilient effect. Effective *RpXDH* silencing in the AM was observed only at the later time point (Supplementary Figure 5B). The abovementioned similar phenotypes obtained with *RpNOX5* or *RpXDH* silencing led us to hypothesize that these gene products could influence each other’s function at the transcriptional level. However, a complex pattern appeared when the effect of *RpXDH* or *RpNOX5* silencing on the expression of the other gene was evaluated; in unfed insects, the silencing of *RpXDH* 6 days beforehand markedly reduced *RpNOX5* expression, and *RpNOX5* silencing also resulted in a slight (non-significant) trend toward an increase in *RpXDH* mRNA levels (Figure 6). On the other hand, at the later time point (4 days ABM), *RpNOX5*-silenced insects showed reduced *RpXDH* levels, while in the *RpXDH*-silenced insects, the *RpNOX5* levels seemed to have returned to control levels (Figure 6).

## Discussion

In several animal models, including insects that are important vectors of human disease, both reactive oxygen and nitrogen species have been implicated as major players in gut immunity. These molecules are known to control both pathogenic and indigenous microbiota and are often described as killing microbes directly or together with other immune mechanisms, such as the insect complement-like protein thioester containing protein (TEP-1) (Oliveira et al., 2012). Here, we studied two enzymes involved in redox balance, the ROS-producing NOX5 enzyme (Bánfi et al., 2001) and an antioxidant enzyme, XDH, which produces the main endogenous ROS-scavenging low-molecular-weight antioxidant in insects (Hilliker et al., 1992; Souza et al., 1997). Our results indicate that urate is an essential signal in the regulation of blood digestion in *R. prolixus* through a mode of action that involves crosstalk between NOX5 and XDH.

Peristaltic contractions move the food along functionally distinct segments of the digestive apparatus and therefore are an essential component of digestive physiology, controlling in this way the pace of digestion. Peristalsis is regulated by neuropeptides in insects (Barrett et al., 1993; Onken et al., 2004; Brugge et al., 2008; Nozawa et al., 2009; LaJeunesse et al., 2010; Villalobos-Sambucaro et al., 2015). In the *D. melanogaster* ovary, NOX5-derived ROS, together with signaling through the proctolin receptor, elevate intracellular calcium, triggering ovarian muscle contraction (Ritsick et al., 2007). A recent report showed a role for NOX5 in the motility of both mammalian smooth muscle and the *R. prolixus* gut, mediated by calcium-calmodulin and endoplasmic reticulum–regulated mechanisms (Montezano et al., 2018).

Here, we show that, in addition to inhibiting peristalsis, *RpNOX5* silencing results in overall digestion impairment and lethality. Efficient silencing of *RpNOX5* was transient, being observed just before the insects were fed, and recovery to normal expression levels was attained by 4 days ABM. This result suggests that the action of *RpNOX5* in the control of digestion is an early event, occurring just after the blood meal and generating a resilient effect that cannot be corrected by reestablishing gene expression levels. Regarding the physiological mechanism behind the severity of the phenotype, one possibility is that these global effects may be a consequence of gut peristalsis inhibition, which delays hemoglobin delivery to the PM, eventually impairing oogenesis. However, our results showed that silencing either *RpNOX5* or *RpXDH* similarly affected cell integrity, including the AM, which is the first segment that receives the incoming blood meal. The arrival of the blood meal in the triatomine midgut is accompanied by large characteristic changes in epithelial cells, which are not seen in the tissues from the *RpNOX5*/*RpXDH*-silenced insects, which display a large number of autophagosomes and damaged mitochondria, despite the presence of blood in their midguts. These changes are reminiscent of those observed in the midgut of *R. prolixus* (Billingsley, 1988) and other triatomine hemipterans that were starved for extended periods of time (Azevedo et al., 2009; Rocha et al., 2010). The appearance of the tissues (as well as other aspects of blood digestion evaluated here) suggests that the insects with either *RpNOX5* or *RpXDH* silencing were not able to trigger a signal for blood digestion, despite the presence of a large blood meal, thus being trapped in a “physiologically unfed status.” A fasting state has deleterious effects on mitochondrial health and quality control, which results in the removal of damaged components by autophagy (Liesa and Shirihai, 2013). One limitation of the present study was that autophagy was identified exclusively based on ultrastructural data, and further research using molecular markers for autophagy is needed to clarify this point. This would cause the accumulation of dysfunctional units and an increase in ROS generation, where counterintuitively, *RpNOX5* silencing increased ROS levels in both the anterior and posterior midguts. In line with this scenario, it was shown in this insect that starvation markedly increased mitochondrial ROS production in the gut and that this effect was reversed by blood feeding (Gandara et al., 2016). Therefore, it is possible to speculate that the redox signaling impairment caused by *RpNOX5* or *RpXDH* silencing could lead to increased mitochondrial ROS, decreased urate levels in the hemolymph and nutrient shortage due to inhibition of digestion, which, together, would induce crucial morphological changes that could explain their anticipated deaths.

As ROS production in the gut is involved in the control of indigenous microbiota, alterations in the microbiota might be an alternative explanation for this severe phenotype. However, *RpNOX5* silencing resulted in modest (if any) alterations in the population of the probiotic symbiont. As no other bacterial species were found in the gut (not shown), as in normal symbionts, it seems unlikely that dysbiosis was the cause of the deleterious phenotype.

The role of XDH has already been studied in *Aedes aegypti*, in which XDH silencing resulted in adult mosquito death after a blood meal (Isoe et al., 2017). These researchers interpreted their data as representing the dual role of urate, both as an antioxidant and as the main end-product of nitrogen metabolism. This essential role of XDH in a blood-feeding insect, further highlighted here by the impact of XDH silencing on digestion, has a major physiological implication, as it suggests that nitrogen excretion exerts feedback control over digestive physiology. This is of utmost relevance because proteins account for 85% of the dry-weight composition of vertebrate blood, and therefore, the production of ammonia (and its excretion as urate) is mandatory to allow the use of the carbon skeleton of amino acids for the production of other basic biomolecules (e.g., carbohydrates and lipids) as energy sources and raw material for structural components.

One of the effects of silencing *RpNOX5* that was not obvious was the reduction in urate concentration in the hemolymph. This might be at least in part attributed to the reduction in digestive activity, which could decrease the production of amino acids and then reduce urate formation. However, we also showed that uricase injection can mimic *RpXDH* or *RpNOX5* silencing, which suggests that urate is a necessary signal for maintaining tissue integrity and regulating digestion and proper handling of a blood meal. Considering the findings described above and that the evaluation of the mRNA levels of both enzymes shown here that revealed a complex reciprocal mutual effect between these enzymes, we hypothesize the existence of crosstalk at the transcriptional and posttranscriptional levels between *RpXDH* and *RpNOX5* and their products urate and superoxide.

**FIGURE 7:**
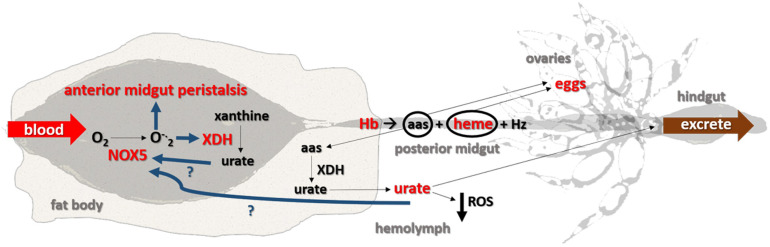
Proposed model. Once blood reaches the anterior midgut, NOX5 is activated, which converts molecular oxygen (O_2_) into superoxide (O^–^_2_), stimulates midgut peristalsis and increases XDH expression. Urate in the midgut may stimulate NOX5 activation. Hemoglobin (Hb) in the anterior midgut is then directed to the posterior midgut, where it is digested into amino acids (aas) and heme and later converted to hemozoin (Hz) to be excreted. Amino acids and heme reach the hemolymph and are then delivered to the ovaries to produce eggs. Some of the amino acids derived from a blood meal are converted into urate in the fat body and act as antioxidant in the hemolymph before eventually being excreted as the final byproduct of nitrogen metabolism. Removing urate from the system (by RNAi silencing or pharmacology) impairs redox homeostasis involving XDH and NOX5. The molecules/functions/pathways in red were assessed in this study and increased after a blood meal; the signaling pathways in dark blue are hypothesized and, the components shown in black were obtained from the literature. The organs are not shown into scale.

Taken together, the data obtained here led us to propose that both superoxide produced by NOX5 and urate produced by XDH are signaling molecules with critical roles in the redox regulation of blood digestion in *R. prolixus*. These results are summarized in the scheme presented in Figure 7, which highlights the cellular and molecular mechanisms that are involved in the control of blood digestion and contribute to adapt these insects to hematophagy.

## Data Availability Statement

The original contributions presented in the study are included in the article/Supplementary Material, further inquiries can be directed to the corresponding author/s.

## Ethics Statement

The animal study was reviewed and approved by CAUAP-UFRJ under registry #IBQM 149-9.

## Author Contributions

PO conceived and coordinated the study. AG and PO designed the experiments and wrote the manuscript. AG performed the experiments shown in Figures 1, 2, 3A, 4, 6, and Supplementary Figures 1, 2, 4, 5. FD performed and analyzed the experiment shown in Figure 5A, Supplementary Figure 3, and Supplementary Table 1, and provided technical assistance. PL performed the experiments shown in Figures 5B,C, and provided technical assistance. RS performed and analyzed the experiment shown in Figure 3B. AG, AB, and RM-B performed and analyzed the experiment shown in Figure 3C. All authors analyzed the results and approved the final version of the manuscript.

## Conflict of Interest

The authors declare that the research was conducted in the absence of any commercial or financial relationships that could be construed as a potential conflict of interest.

## Footnotes

1 http://pfam.sanger.ac.uk/
