# Supplementary material for: “Urate and NOX5 Control Blood Digestion in the Hematophagous Insect Rhodnius prolixus”
Source: Front Physiol. 2021 Feb 25;12:633093. doi: 10.3389/fphys.2021.633093 (PMC7947236; doi:10.3389/fphys.2021.633093)
Supplement: Supplementary file 1 [file Data_Sheet_1.PDF]

Supplementary Material

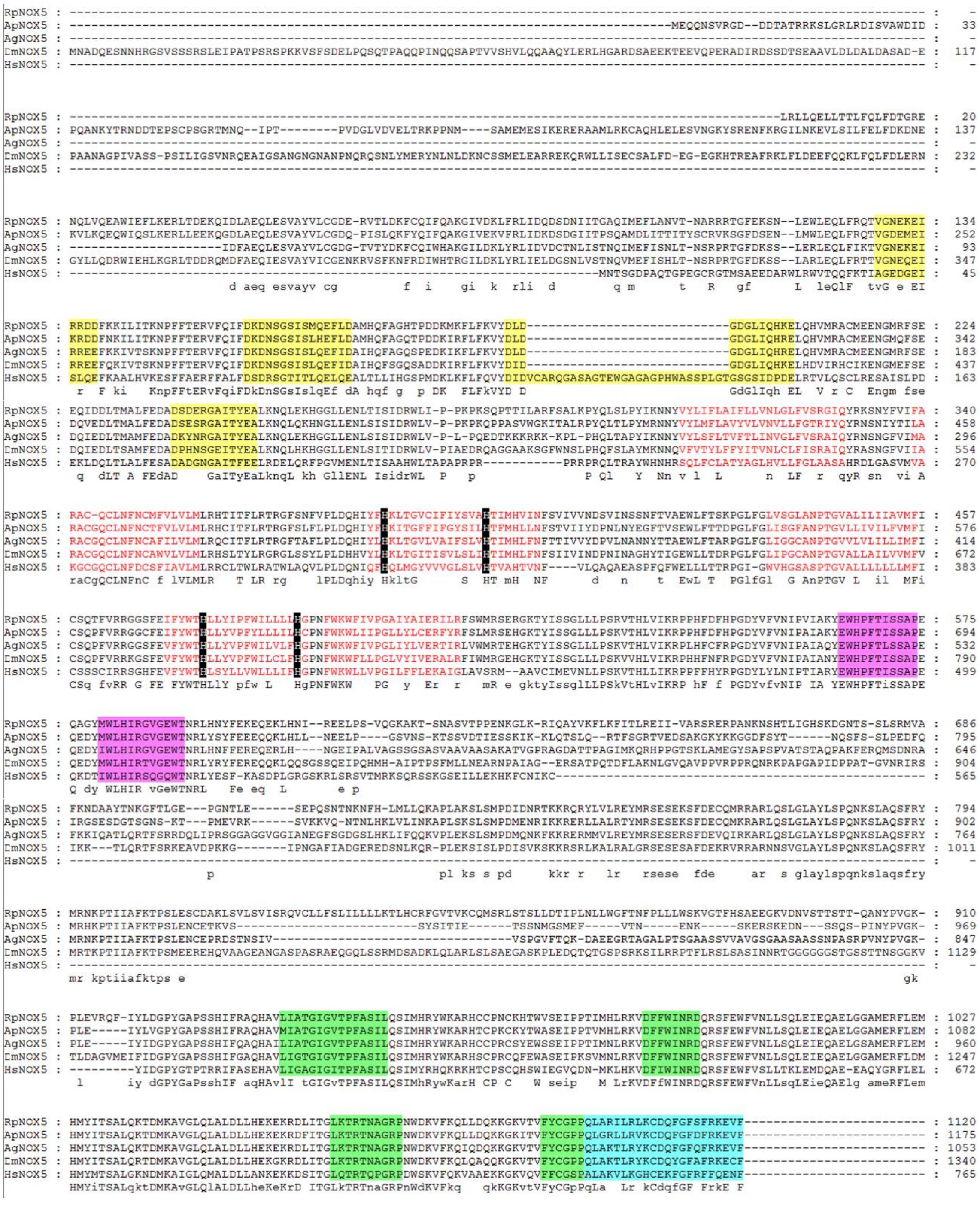

FIGURE S1: Sequence and structural feature of *RpNOX5*. Amino acid sequence alignment of NOX5 orthologs. From top to bottom, NOX5 sequences of *R. prolixus* (RpNOX5), *A. pisum* (ApNOX5), *A. gambiae* (AgNOX5), *D. melanogaster* (DmNOX5) and *H. sapiens* (HsNOX5) are shown. The EF-hand 1-4 calcium-binding sites (yellow), FAD1 and FAD2 (pink), NADPH1-4 (green), C-terminus (blue) and conserved histidine residues (black) are shown in the colors indicated. Transmembrane regions (1-6) are indicated as red letters. Consensus sequences are indicated below the alignment.

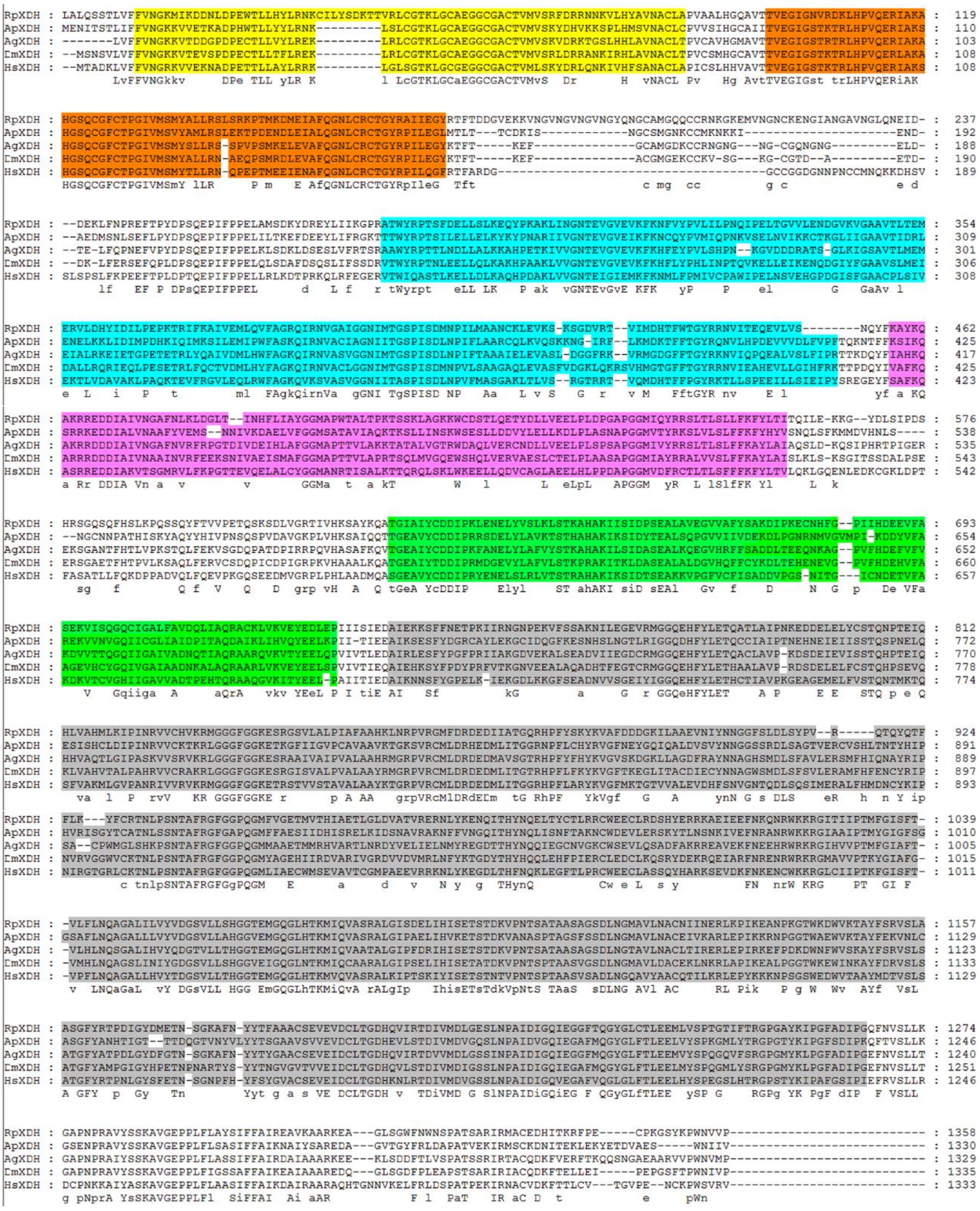

FIGURE S2: Sequence and structural feature of *RpXDH*. Amino acid sequence alignment of XDH orthologs. From top to bottom, XDH sequences of *R. prolixus* (RpXDH), *A. pisum* (ApXDH), *A. gambiae* (AgXDH), *D. melanogaster* (DmXDH) and *H. sapiens* (HsXDH) are shown. Ferredoxin (2Fe-2S iron sulfur cluster-binding domain) (yellow), 2Fe-2S cluster (orange), FAD-binding domain (blue), CO dehydrogenase flavoprotein C-terminus (pink), aldehyde oxidase hammerhead (green) and molybdopterin-binding domain (gray) are shown in the indicated colors. Consensus sequences are indicated below the alignment.

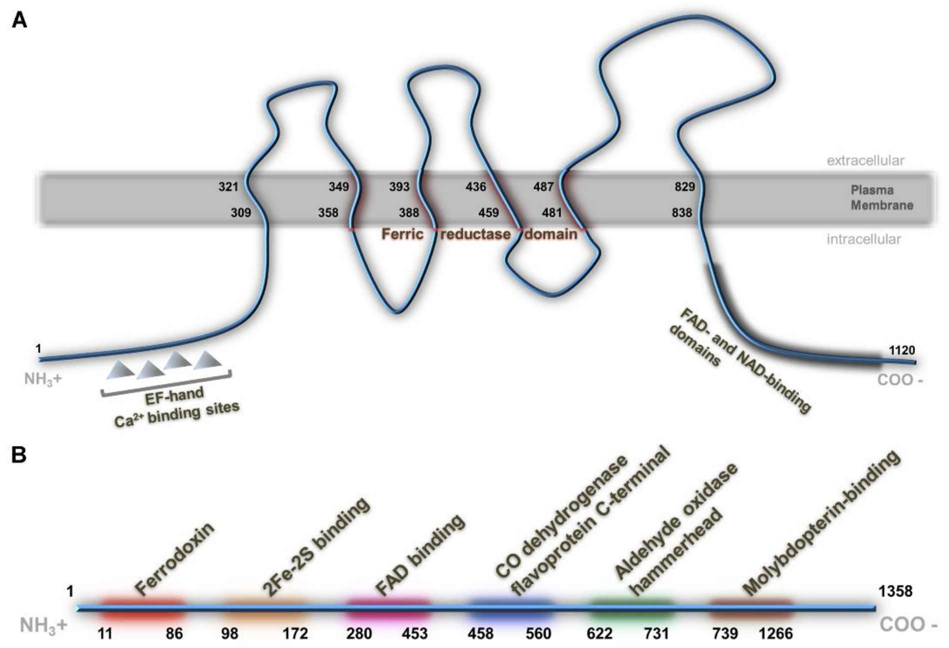

FIGURE S3: Schematic representations of *R. prolixus* NADPH oxidase 5 and xanthine dehydrogenase proteins. A local BLAST search using the cDNA sequences of NOX5 (A) and XDH (B) orthologs as queries was used to identify the NOX5 and XDH sequences in the *R. prolixus* 454 transcriptome and genome databases. The positions of canonical domains were predicted and identified with TMHMM and Pfam.

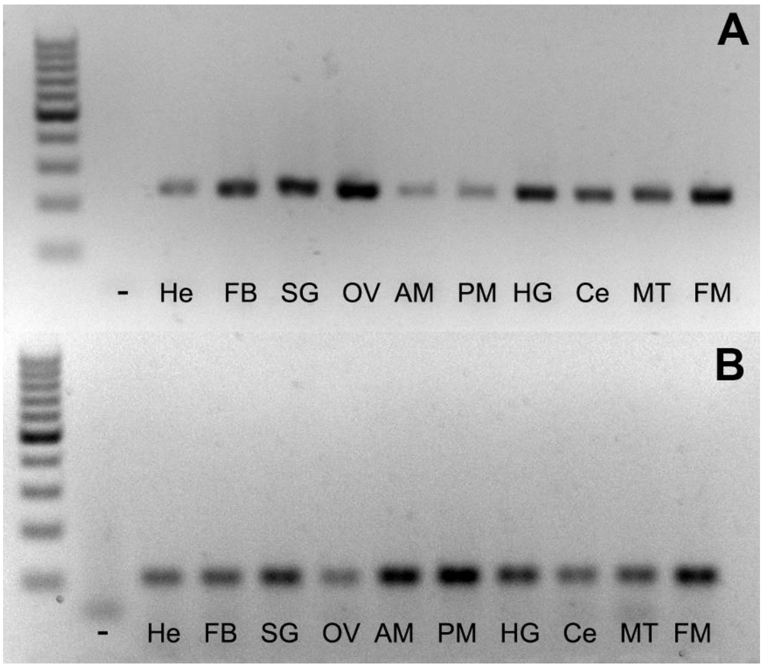

FIGURE S4: Tissue distribution of *RpNOX5* and *RpXDH*. (A) *RpNOX5* and (B) *RpXDH* fragments were RT-PCR-amplified from cDNA samples from heart (He), fat body (FB), salivary glands (SG), ovary (OV), anterior midgut (AM), posterior midgut (PM), hindgut (HG), cerebrum (Ce), Malpighian tubules (MT) and flight muscle (FM) and were separated by 2% agarose gel electrophoresis. (-, no cDNA).

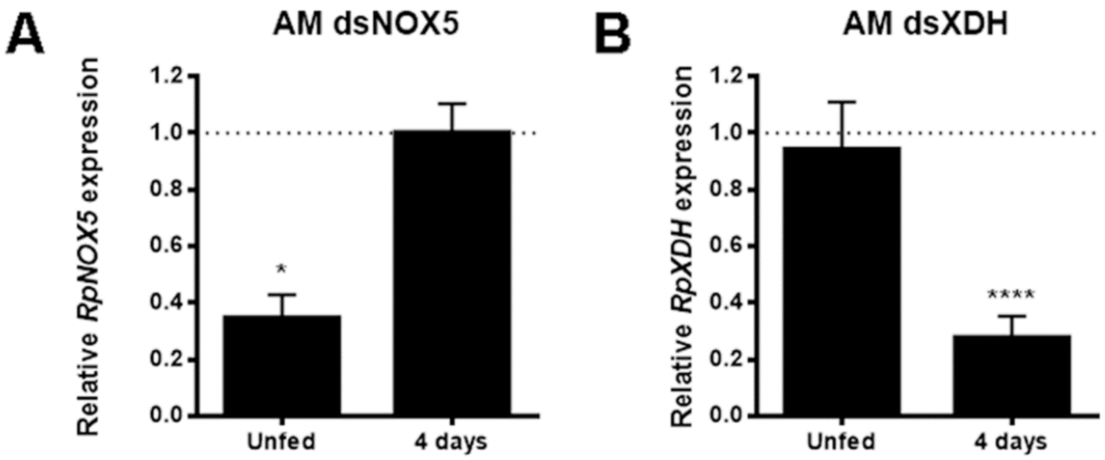

FIGURE S5: Confirmation of *RpNOX5 and RpXDH* expression silencing. (A, B) *RpNOX5* and *RpXDH* expression levels in the anterior midgut (AM). qPCR assays were performed with individual tissues dissected 6 days after dsRNA injection before a blood meal (unfed) or 4 days ABM. Gene expression levels were normalized to the levels found in the dsMal-injected animals (7-26 insects per condition). * $P < 0.05$, **** $P < 0.0001$, compared with dsMal-injected animals. Unpaired t-test. The data represent the mean ± SEM.

Gene	Forward	Reverse
XDHRt	5’-TTCACAAGGGCAGTGTATCG-3’	5’-CACCCATCCTGACTTCACCT-3’
NOX5Rt	5’-GCATGGTGGCGTTTAAGAAT-3’	5’-AACGGAGCTTTTTGAAGCAA-3’
EF1Rt	5’-GATTCCACTGAACCGCCTTA-3’	5’-GCCGGGTTATATCCGATTTT-3’
NOX5Ds1	5’-CAGACTGTCGGCAATGAAAA-3’	5’-GTTTTGGCGGTATCAACCAG-3’
XDHDs1	5’-GTCACATGGAGGCACAGAGA-3’	5’-TCTCACCGAGATCCATCACA-3’
NOX5Ds2	5’-TAATACGACTCACTATAGGGCAGACTGTCGGCAATGAAAA-3’	5’-TAATACGACTCACTATAGGGGTTTTGGCGGTATCAACCAG-3’
XDHDs2	5’-TAATACGACTCACTATAGGGGTCACATGGAGGCACAGAGA-3’	5’-TAATACGACTCACTATAGGGTCTCACCGAGATCCATCACA-3’

TABLE S1: Primer sequences used in this study.
